# Correlation between Allergic Rhinitis and Laryngopharyngeal Reflux

**DOI:** 10.1155/2018/2951928

**Published:** 2018-03-22

**Authors:** Sami Alharethy, Abdulsalam Baqays, Tamer A. Mesallam, Falah Syouri, Mawaheb Al Wedami, Turki Aldrees, Alhanouf AlQabbani

**Affiliations:** ^1^Division of Facial Plastic Surgery, Department of Otolaryngology, Head & Neck Surgery, King Abdulaziz University Hospital, King Saud University, P.O. Box 245, Riyadh 11411, Saudi Arabia; ^2^Department of Otolaryngology, Head & Neck Surgery, King Abdulaziz University Hospital, King Saud University, Riyadh, Saudi Arabia; ^3^Research Chair of Voice, Swallowing, and Communications Disorders, ORL Department, College of Medicine, King Saud University, Riyadh, Saudi Arabia; ^4^Otolaryngology-Head & Neck Department, Shiekh Khalifa Medical City, Abu Dhabi, UAE; ^5^Department of Otolaryngology, Head and Neck Surgery, Prince Sattam bin Abdulaziz University, Alkharj, Saudi Arabia; ^6^Department of Otolaryngology, Head & Neck Surgery, Prince Sultan Medical Military City, Riyadh, Saudi Arabia

## Abstract

**Background and Objectives:**

Laryngopharyngeal reflux (LPR) exhibits nonspecific clinical presentations, and these symptoms may be associated with other conditions such as allergies, including allergic rhinitis and laryngitis. However, there is a gap in the literature regarding the correlation of laryngopharyngeal reflux with allergic rhinitis/laryngitis. Hence, the aim of this study is to explore the correlation between these two conditions.

**Patients and Methods:**

A total of 126 patients with suggestive manifestations of laryngopharyngeal reflux were included in this study. Patients were classified into LPR positive and negative groups based on the results of a 24-hour oropharyngeal pH monitoring system while allergic rhinitis status was assessed with the score for allergic rhinitis (SFAR).

**The results of the two groups were compared regarding the SFAR score. Correlation between the pH results and SFAR score was explored. Results:**

The LPR positive group demonstrated significantly higher SFAR scores compared to the negative LPR group (*p* < 0.0001). In addition, the Ryan score was significantly correlated with the* SFAR total score and its symptomatology-related items (r ranged between 0.35 and 0.5). Conclusion.* It seems that laryngopharyngeal reflux increases patients' self-rating of allergic manifestations. It appears that there is an association between laryngopharyngeal reflux and allergic rhinitis/laryngitis.

## 1. Introduction

During the last decades, interest in exploring gastric reflux and understanding its comorbidity has increased. Asher Winkelstein discovered gastroesophageal reflux disease (GERD) in 1935, and it was clinically diagnosed by the presence of typical symptoms such as heartburn and acidic regurgitation [1]. Interestingly, otolaryngologists found that some patients presented with no specific symptoms arising from the upper aerodigestive tract with substantial evidence of acidic reflux sequelae despite a lack of typical GERD symptoms [2]. A new era of interest in the field of GERD research was subsequently established to answer the following questions: what is laryngopharyngeal reflux (LPR)? What are the factors that distinguish LPR from GERD? How can we diagnose LPR? And finally, what is the perfect plan to manage LPR [3]?

Reflux of gastric contents into the upper aerodigestive tract despite the absence of heartburn and regurgitation is what defines LPR [4]. As stated in the literature, there are debates regarding whether to consider it as an atypical presentation of GERD or an entirely different disease entity known as LPR [5, 6]. LPR and GERD can be differentiated; heartburn and acidic regurgitation that commonly occur at night and frequently in the supine position in addition to classical sequelae that demonstrate the presence of esophagitis as detected by endoscopy or pH monitoring systems indicate a diagnosis of GERD [4, 7]. In contrast, the diagnosis of the LPR is a complex process. The diagnosis encompasses cumulative results of clinical interviews and investigations and even challenging treatment methods [7–9]. Based on clinical history, LPR presents with ambiguous symptoms such as hoarseness, throat clearing, and globus pharyngeus [7, 10]. Thus, the determination of the precise prevalence of LPR is a challenge.

LPR was initially reported in 1968 by Cherry and Margulies [2]. Since that time, the association of LPR with other medical conditions has been recognized. This association encompasses chronic pharyngitis [11], obstructive sleep apnea [12], chronic rhinosinusitis [13–15], and asthma [16]. Furthermore, the awareness of LPR as an airway disease has grown [3, 17]. However, one of the most common airway diseases in the world is allergic rhinitis (AR). AR is defined as an inflammatory process that occurs in the nasal mucosa that is stimulated by exposure to allergens [18]. It is a common disease all over the world. The prevalence of AR continues to increase worldwide. AR usually occurs with other allergic diseases. A report released by the World Health Organization reveals that up to 40% of the world's population has one or more allergic conditions [19]. Extensive studies are trying to elucidate the prevalence and risk factors of AR. Unfortunately, no specific estimation for AR among the world's population has been released. One trial that estimated the prevalence of the disease demonstrated that 21% of 3,001 French people were diagnosed with AR. Abdul Rahman et al. conducted a survey on 501 Middle Eastern individuals and found that 10% of the respondents were diagnosed with AR [20]. This disease can occur in all age groups. The worldwide concern came from the finding that AR is a common risk factor for several airway conditions [21]. AR can manifest as nasal pruritus, sneezing, rhinorrhea, postnasal drip, nasal congestion, sore throat, globus sensation, throat cleaning, and dysphonia [22, 23]. Hence, LPR and AR/AL are somehow interrelated to each other in terms of clinical presentations. In addition there is a gap in the available knowledge to identify the relationship between these two conditions. In this study, we are aiming to explore the associations between the LPR and AR/AL.

## 2. Patients and Methods

This study is considered observational and analytical and was carried out in the reflux clinic at the otolaryngology department. A total of one hundred twenty-six patients who presented to the clinic with symptoms suggestive of LPR were included in the study. Patients filled up the SFAR questionnaire to assess their allergic rhinitis status. In addition, all patients underwent 24-hour oropharyngeal pH monitoring to detect the presence of LPR.

### 2.1. 24-Hour Oropharyngeal pH Monitoring

Based on the results of the 24-hour oropharyngeal Dx-pH probe system (Restech Corp., San Diego, CA, USA), a diagnosis of LPR was confirmed in the participants. As part of the standard protocol for this instrument, patients were instructed to maintain their usual daily activity. Patients recorded meal times and recumbent position times in a diary, as per the instructions of the study team. Analyses were performed with the software provided with machine based on the recorded information. In this study, we depended on pH levels of 5.5 and 5 as the thresholds for diagnosing LPR in an upright and supine position, respectively. As a standard protocol of pH measurement, meal times were excluded from the analysis to achieve an accurate result. The Ryan score was automatically produced by the system. It is a composite score that encompasses three main parameters, which are the number of reflux episodes, the duration of the longest reflux episode, and the percentage of time below the predetermined pH threshold. If the Ryan score was greater than 9.41 in the upright position or 6.80 in the supine position, then a diagnosis of LPR was confirmed.

### 2.2. SFAR Rating

All patients in the study were instructed to complete the Arabic version of SFAR (supplementary materials (available here)) [24] before being admitted to the clinic. Patients were asked to complete the questionnaire precisely according to their current condition. Patients rated their allergic rhinitis-related problems in the SFAR after detailed instructions on how to respond to items in the questionnaire. Ratings were documented according to the scoring system suggested by the authors, and a total score was given for each patient.* Based on the SFAR scoring system, patients with a total score of 7 or more are considered for AR diagnosis.*

### 2.3. Statistical Analysis

Patients in the study were divided into positive and negative LPR groups according to results of the 24-hour oropharyngeal pH monitoring. Comparisons were made between the positive and negative LPR groups regarding the results of the SFAR questionnaire.* In addition, a correlation was conducted between the results of 24-hour pH monitoring and both the individual items and total SFAR scores. Also the frequency of positive AR diagnosis has been compared between the positive and negative LPR groups.* Nonparametric statistical analyses were applied in this study. Spearman's correlation coefficient was used to test the correlation between pH results and SFAR results while the Mann–Whitney test was used for the comparison of the SFAR rating results among the positive and negative LPR groups.* Chi Square test was used to examine the difference between the negative and positive LPR groups regarding the frequency distribution of AR diagnosis.* The level of significance was set as *p* < 0.05. The Statistical Package for the Social Sciences, Version 22 (SPSS Inc, Chicago, IL, USA), was used for all statistical analyses.

## 3. Results

The study included 126 patients (70 females and 56 males) with a mean age of 39.4 ± 21.2 years. Oropharyngeal 24-hour pH monitoring was completed in all patients in the study and, according to the results, patients were classified into positive and negative laryngopharyngeal reflux groups. Interestingly, there were 63 patients with positive laryngopharyngeal reflux (positive LPR group) and 63 patients with negative laryngopharyngeal reflux (negative LPR group). All patients completed the SFAR questionnaire for allergic rhinitis, and the ratings ranged between 0 and 16 with a mean score of 8 ± 4.24. Based on the SFAR scoring system there were 84 patients with positive AR diagnosis while 42 patients were with negative AR diagnosis. Among the positive AR patients, 54 were in the positive LPR representing 85% of total group number. On the other hand there were 30 patients with positive AR diagnosis in the negative LPR group representing 48% of the total group number (Table 2). Upon comparing the positive and negative LPR groups regarding the SFAR score, there was a significant difference between the two groups, with significantly higher total scores reported in the positive group for the total SFAR score and items 1, 2, and 5 (Table 1). Also there was significantly higher frequency of positive AR diagnosis reported in the positive LPR group compared to the negative LPR group (Table 2). On the other hand, there was a significant positive correlation between the pH Ryan score and the total SFAR score as well as items 1 and 5 of the SFAR (Table 3).

## 4. Discussion

The larynx is situated in a crucial location and is believed to be the connecting structure between the upper and lower airway systems. The uniformity of microscopic structures along the whole respiratory system indicates that these two systems are interrelating units that function for each other. Based on this finding, Krouse proposed that the presence or exacerbation of a disease process in one part of the airway is likely going to produce effects in the entire respiratory system simultaneously [25]. Keller presented an interesting result in support of this hypothesis. He reported that 86% of his asthmatic population had concurrent nasal symptoms [26]. Moreover, the role of allergy in laryngeal irritation and voice problems has been an interest of many researchers. Allergic rhinitis and its effect on nasal mucosa could lead to similar effects on laryngeal mucosa including edema, excessive mucous secretion, and congestion [27, 28]. Hence, the aim of this study was to explore associations between AR/AL and LPR.

Half of our study participants who presented with suggestive symptoms of LPR were grouped objectively based on the 24-hour oropharyngeal pH monitoring system results into positive and negative LPR groups. Ayazi et al. presented this diagnostic technique in 2009 [29]. Several instrumental and noninstrumental techniques are available for the diagnosis of LPR. For example, Amin et al. performed throat biopsies on all patients and examined the samples under an electronic microscope to detect the dilatation of intercellular spaces (DIS) in the oropharyngeal mucosa. He found that DIS is common in patients with LPR in his group. Hence, he concluded that DIS is more sensitive and specific for the diagnosis of LPR [30]. However, a smaller sample size was used, which was appropriate for his project, but the application of this technique in a larger population is not feasible. Additionally, the positive LPR and control groups were identified with subjective methods such clinical history, pharyngolaryngoscopy, and endoscopy of the upper gastrointestinal tracts without the use of any pH monitoring systems as an objective assessment. Lastly, Amin's method of diagnosis is more invasive, time consuming, and expensive than pH monitoring techniques.

However, the noninstrumental methods for the diagnosis of LPR encompass the reflux symptoms index (RSI) and the reflux finding score (RFS). Both of these scores were developed by Belafsky et al. [10, 31]. Although RSI has more sensitivity and specificity than RFS in detecting LPR as reported by Musser et al. [32] and Mesallam et al. [33], none of them can be used as the main instrument for LPR diagnosis. LPR manifested clinically with dysphonia, frequent throat clearing, and globus sensation. Interestingly, AR/AL can present with similar manifestations, as reported by Krouse and Altman [34]. Overlapping of clinical presentations between both conditions has played a role in the misdirection of clinicians or scientists to obtain precise diagnoses. Randhawa et al. studied 15 persons to identify a correlation between dysphonia and allergy and LPR. In their project, RSI and RFS were used to diagnose LPR, and both the skin prick test (SPT) and nitrous oxide (NO) were used to assess allergy status. Randhawa et al. concluded that there were no significant differences between allergy and LPR [22].* However, the use of a smaller study population beside the nonobjective LPR diagnostic method may mask the accuracy of the conclusion.*

In this study we included 126 patients who presented to the clinic with clinical manifestations suggestive of LPR. SFAR was used to evaluate and diagnose AR in addition to 24-hour pH monitoring for the diagnosis of LPR. We found a significant positive correlation between Ryan score of pH monitoring and total SFAR score. Also, significant positive correlation was reported between Ryan score and both item numbers 1 and 5 in SFAR questionnaire. As could be understood from the SFAR questionnaire, item 1 is the main item considering symptoms such as sneezing, runny nose, and blocked nose while item 5 is testing the patient's self-perception of allergic condition. These findings confirm the correlation between the LPR and AR presentation. Additionally, both items 1 and 5 as well as total SFAR scores were significantly higher in the positive LPR group compared to the negative LPR group. Although, in their study, Eren et al. [35] did not find a significant difference between positive and negative allergic rhinitis groups regarding reflux symptoms and scores, they concluded that the presence of thick endolaryngeal secretion in LPR patients should raise the suspicion of allergic rhinitis/laryngitis. The diversity of results found in the current study and previously published studies can be explained by the different approaches used to define the study groups. In our study, we used an objective diagnostic tool to identify LPR (pH monitoring) with a subjective allergy test (SFAR). Other studies used a more objective test in the diagnosis of allergic conditions (skin prick test) and subjective testing for LPR (RSI and RFS).* In the current study, the frequency of positive AR diagnosis was also significantly higher in the positive LPR group compared to the negative LPR one. This finding in addition to the abovementioned positive correlation finding adds more support to the hypothesis of having a relationship between LPR and AR/AR regarding the clinical presentation.* One of the limitations of the current study is the absence of an objective test for allergy. However, despite being used in many allergy tests, there is a still debate regarding the accuracy of the skin prick test in diagnosing allergies including allergic rhinitis and there is no consensus among researchers about its validity [36–39].

## 5. Conclusion

Dysphonia, frequent throat cleaning, and a globus sensation are common presentations of LPR and allergic rhinitis/laryngitis. It appears that there is an association between LPR and allergic rhinitis/laryngitis. LPR can be considered a cofactor in increasing patients' self-perception of allergic problems.

## Figures and Tables

**Table 1:** Comparison between the positive and negative LPR groups regarding SFAR score results.

SFAR	LPR positive group	LPR negative group	*p*
	Mean (SD)	Mean (SD)	
Item 1	2.21 (0.93)	1.26 (1.15)	<0.0001
Item 2	0.86 (0.99)	0.47 (0.85)	0.023
Item 3	1.21 (0.59)	1.11 (0.74)	0.43
Item 4	1.63 (0.73)	1.52 (0.85)	0.43
Item 5	1.68 (0.73)	0.6 (0.92)	<0.0001
Item 6	0.32 (0.73)	0.19 (0.59)	0.28
Item 7	0.46 (0.5)	0.38 (0.48)	0.37
Item 8	1.33 (0.95)	1.26 (0.97)	0.71

Total	9.69 (3.69)	6.82 (4.27)	<0.0001

**Table 2:** Comparison between the positive and negative LPR groups regarding frequency of positive AR diagnosis in the two groups.

Groups	LPR positive (*n* = 63)	LPR negative (*n* = 63)	*p*
AR positive *n* (%)	54 (85%)	30 (48%)	0.002
AR negative *n* (%)	9 (15%)	33 (52%)	

**Table 3:** Correlation between the SFAR score (item and total scores) and Ryan score.

	Correlation		
	SFAR		Ryan score
Spearman's rho	Item 1	Correlation coefficient	0.464^*∗∗*^
		Sig. (2-tailed)	0.000
		*N*	126
	Item 2	Correlation coefficient	0.158
		Sig. (2-tailed)	0.076
		*N*	126
	Item 3	Correlation coefficient	0.119
		Sig. (2-tailed)	0.184
		*N*	126
	Item 4	Correlation coefficient	0.010
		Sig. (2-tailed)	0.911
		*N*	126
	Item 5	Correlation coefficient	0.503^*∗∗*^
		Sig. (2-tailed)	0.000
		*N*	126
	Item 6	Correlation coefficient	0.132
		Sig. (2-tailed)	0.141
		*N*	126
	Item 7	Correlation coefficient	0.088
		Sig. (2-tailed)	0.325
		*N*	126
	Item 8	Correlation coefficient	0.075
		Sig. (2-tailed)	0.402
		*N*	126
	Total	Correlation coefficient	0.354^*∗∗*^
		Sig. (2-tailed)	0.000
		*N*	126

^*∗∗*^Correlation is significant at 0.01.

## Supplementary Materials

Supplementary Materials Standardized questionnaire for SFAR assessment in English and Arabic.
