# Supplementary material for: Correlation between Allergic Rhinitis and Laryngopharyngeal Reflux
Source: Biomed Res Int. 2018 Mar 22;2018:2951928. doi: 10.1155/2018/2951928 (PMC5885348; doi:10.1155/2018/2951928)
Supplement: Supplementary Materials — Standardized questionnaire for SFAR assessment in English and Arabic. [file 2951928.f1.zip › 2951928.f1/2951928.f1.docx]

**Appendix I**

**إستبيان معدل حساسية الأنف**

**الرجاء وضع علامة ( صح ) أمام الإجابة المناسبة:**

1. **خلال السنة الماضية بغض النظر عن نزلات البرد والزكام,هل عانيت من المشاكل التالية:**

- العطاس نعم لا
- سيلان الأنف نعم لا
- إنسداد الأنف نعم لا

1. **في حالة كانت الإجابة بنعم,خلال السنة الماضية هل كانت مشكلة الحكة وتدميع العينين مصاحبة لمشكلة الأنف؟**

نعم لا

1. **في أي من شهور السنة الماضية أو من فصول السنة حدثت مشاكل الأنف؟**

يناير فبراير مارس أبريل

مايو يونيو يوليو أغسطس

سبتمبر أكتوبر نوفمبر ديسمبر

الشتاء الربيع الصيف الخريف

1. **ماهي العوامل التي قد تحفز من زيادة مشاكل الأنف لديك؟**

غبار المنزل حشرة غبار المنزل حبوب اللقاح

الحيوانات (القطط,الكلاب...الخ) أخرى (الرجاء التحديد )

1. **هل تعتقد أنك تعاني من الحساسية ؟**

نعم لا

1. **هل سبق أن أجريت إختبار للحساسية و إختبار الجلد وتحليل الدم؟**

نعم لا

في حالة كانت الإجابة بنعم,فهل كانت نتيجة التحليل؟

إيجابية سلبية

1. **هل تم تشخيصك من قبل الطبيب بحساسية الصدر والأنف أو بحساسية الجلد؟**

نعم لا

1. **هل يعاني أي فرد من أسرتك من حساسية الصدر والأنف أو بحساسية الجلد؟**

نعم لا

في حالة كانت الإجابة بنعم,الرجاء تحديد فرد الأسرة المصاب وماهي المشكلة؟

الأب حساسية الصدر حساسية الأنف حساسية الجلد

الأم حساسية الصدر حساسية الأنف حساسية الجلد

الأبناء حساسية الصدر حساسية الأنف حساسية الجلد
