# Supplementary material for: Correlation between Allergic Rhinitis and Laryngopharyngeal Reflux
Source: Biomed Res Int. 2018 Mar 22;2018:2951928. doi: 10.1155/2018/2951928 (PMC5885348; doi:10.1155/2018/2951928)
Supplement: Supplementary Materials — Standardized questionnaire for SFAR assessment in English and Arabic. [file 2951928.f1.zip › 2951928.f1/2951928.f2.docx]

Appendix I

Standardized questionnaire for SFAR assessment

1. In the past 12 months, have you had a problem apart from cold or ﬂu with (please tick appropriate cases(s)) :

Sneezing No □ Yes □

Runny nose No □ Yes □

Blocked nose No □ Yes □

If YES (at least one nose problem):

1. In the past 12 months, has this nose problem been accompanied by itchy-watery eyes?

No □ Yes □

1. In which of the past 12 months (or in which season) did this nose problem occur?

Jan □ Feb □ Mar □ Apr □ May □

June □ July □ Aug □ Sept □ Oct □

Nov □ Dec □

(or alternatively)

Winter □ Spring □ Summer □ Autumn □

1. What trigger factors provoke or increase your nose problem?

House dust □

House dust mites □

Pollens □

Animal (cat, dogs…) □

Others (please specify) ___________________________________________________

1. Do you think to be allergic?

No □ Yes □

1. Have you already been tested for allergy (SPT, IgE)?

No □ Yes □

If YES:

6a What was the result?

Positive □ Negative □

1. Has a doctor already diagnosed that you suffer/suffered from asthma, eczema or allergic rhinitis?

No □ Yes □

1. Is any member of your family suffering from asthma, eczema or allergic rhinitis?

No □ Yes □

YES: Who and what disease? (please tick appropriate cases(s)):

Father Asthma □ Eczema □ Allergic rhinitis □

Mother Asthma □ Eczema □ Allergic rhinitis □

Siblings Asthma □ Eczema □ Allergic rhinitis □
